# Whole genome bisulfite sequencing reveals unique adaptations to high-altitude environments in Tibetan chickens

**DOI:** 10.1371/journal.pone.0193597

**Published:** 2018-03-21

**Authors:** Zengrong Zhang, Huarui Du, Lijun Bai, Chaowu Yang, Qingyun Li, Xiaocheng Li, Mohan Qiu, Chunlin Yu, Zongrong Jiang, Xiaoyu Jiang, Lan Liu, Chenming Hu, Bo Xia, Xia Xiong, Xiaoyan Song, Xiaosong Jiang

**Affiliations:** 1 Sichuan Animal Science Academy, Chengdu, Sichuan, China; 2 Animal Breeding and Genetics key Laboratory of Sichuan Province, Chengdu, Sichuan, China; 3 BGI-Shenzhen, Shenzhen, China; 4 Ganzi Animal Science institute, Ganzi Tibetan Autonomous Prefecture, Kangding, Sichuan, China; Inc, UNITED STATES

## Abstract

**Background:**

Tibetan chickens living at high altitudes show specific adaptations to high-altitude conditions, but the epigenetic modifications associated with these adaptations have not been characterized.

**Results:**

We investigated the genome-wide DNA methylation patterns in Tibetan chicken blood by using whole genome bisulfite sequencing. Generally, Tibetan chickens exhibited analogous methylation patterns to that of lowland chickens. A total of 3.92% of genomic cytosines were methylcytosines and 51.22% of cytosines in CG contexts were methylated, which was less than those in lowland chicken (55.69%). Moreover, the base adjacent to the methylcytosines of mCHGs in Tibetan chickens had a preference for T, which was different from that in lowland chickens. In Tibetan chickens, the methylation levels in the promoter were relatively low, while the gene body was also maintained in a hypomethylated state. DNA methylation levels in regions upstream of the transcription start site of genes were negatively correlated with the level of gene expression, and DNA methylation of gene body regions was also negatively related to gene expression.

**Conclusions:**

We generated the genome-wide DNA methylation patterns in Tibetan chickens and our results will be helpful for future epigenetic studies related to adaptations to high-altitude conditions.

## Introduction

DNA methylation is a crucial epigenetic modification that plays a vital role in genomic imprinting [1], transcriptional repression [2], and chromatin activation [3]. In recent years, we have gained knowledge regarding the association between DNA methylation and cellular differentiation, development, and disease. However, little information is available concerning DNA methylation modifications when organisms are in long-term, extreme environments.

Environmental factors influence through genetic and epigenetic mechanisms [4,5]. Several studies have attempted to establish a relationship between environmental factors and DNA methylation in humans. It was reported that reduced global DNA methylation in whole blood was related to exposure to ambient air pollution in the homes of adults [6]. In malignant cells, airborne benzene induces a significant decrease in the methylation of long interspersed element-1 (LINE-1) and AluI repetitive elements, and increasing airborne benzene levels can cause hypermethylation in *P15* and hypomethylation in melanoma-associated-antiGEn homolog-1 (*MAGE-1*) [7]. The average level of methylation in *P16* was increased in patients with benzene poisoning compared with a control group, while no change was observed in the *P15* methylation [8]. Korea et al. revealed that most organochlorine pesticides were inversely and significantly correlated to the methylation of Alu elements [9]. In pregnant women, lead exposure was inversely related to genomic DNA methylation patterns in white blood cells [10]. Moreover, based on the epigenetic inheritance mechanism, adaptive traits that result from environmental factors can be transferred to the next generation. For instance, the environments containing endocrine-disrupting chemicals can affect the germline and promote disease in offspring via DNA methylation [11].

Above researches showed that environmental conditions could induce DNA methylation alterations and influence disease, we explored whether DNA methylation is associated with the adaptation of farm animals to hypoxia and high-dose ultraviolet radiation in high-altitude environments. The Tibetan chicken, which lives in a high-altitude environment, has a smaller body, lower heart rate, higher turnover of cells in the spleen, and higher erythrocyte volume than low-altitude chickens. Previous research showed that humans relocating to high-altitudes may undergo acute mountain sickness, high-altitude pulmonary edema, and high-altitude cerebral edema [12]. However, the Tibetan chicken is well-adapted to the low-oxygen, high-altitude environment. It also has a long life expectancy and has a high reproduction capacity [13]. Therefore, an investigation into the genome-wide DNA methylation patterns in Tibetan chickens may lead to an understanding of the adaptability of these chickens and may provide ideas for the prevention and treatment of mountain sickness and other hypoxia-related diseases in humans.

In this study, we performed whole-genome bisulfite sequencing (WGBS) on Tibetan chicken blood to analyze the global DNA methylation patterns. The DNA methylome distribution in the Tibetan chicken genome was shown for the first time. Our results will provide an important resource for exploring low-oxygen adaptation mechanisms in high-altitude areas.

## Materials and methods

### Ethics statement

All procedures conducted with the chickens were performed in accordance with relevant guidelines and regulations and were approved by the Science and Technology Department of the Sichuan Province and the Animal Care and Use Committee of the Sichuan Animal Science Academy. No associated permit number was required since commercial animal sampling was approved. All efforts were made to minimize animal suffering.

### Animals

In this study, three Tibetan chickens were obtained from Xiangcheng County in the Ganzi Tibetan Autonomous Prefecture. This location is approximately 3500 meters above sea level. A 2ml disposable syringe was used to extract 1ml of venous blood from the venous veins of each chicken. The chickens were sterilized with medical alcohol before the blood was collected and there was no slaughtering. Blood samples were collected and stored at -20°C for bisulfite sequencing. Total genomic DNA was isolated from the blood with the use of a TIANamp Genomic DNA Kit (Tiangen Biotech, Beijing, China).

### MethylC-Seq library construction and sequencing

DNA was fragmented with a sonicator (Sonics & Materials) to a mean size of approximately 250 bp, followed by blunt ending, the 3′-end addition of dA, and adapter ligation, in which Illumina methylated adapters were used according to the manufacturer’s instructions. The bisulfite conversion of Tibetan chicken DNA was carried out using the ZYMO EZ DNA Methylation-Gold Kit (Zymo Research, Irvine, CA, USA) and amplified via PCR with 12 cycles. Ultra high-throughput paired-end sequencing was performed by the Illumina Genetic Analyzer (GA2), using manufacturer’s instructions. The Illumina base-calling pipeline (SolexaPipeline-1.0) was used to process raw GA sequencing data.

### Data filtering

Data filtering was performed by the elimination of contaminating DNA and low-quality reads from the raw reads. Low-quality reads include three types, and the read which accord with one of them will be removed: 1) Contain adaptor sequence; 2) N base number over 10%; 3) The number of base whose quality less than 20 over 10% was trimmed. Only clean data were used for the further analyses.

### Read alignment

On the forward read of each read pair, observed cytosines were replaced with adenines and the observed guanines were replaced with adenines on the reverse read of each read pair. The “alignment form” reads were then mapped to the “alignment form” Gallus_gallus reference genome by the Short Oligonucleotide Analysis Package (SOAP) aligner [14]. Each hit, including a single place with a minimum number of mismatches and a clear operation chain, was defined as an unambiguous alignment (uniquely mapped reads) and was used for ascertainment of methyl cytosine. The copy numbers in the local region were estimated by calculating the uniquely mapped reads.

### Estimating methylation levels

Methylation level was determined by dividing the number of reads covering each mC by the total number of reads covering that cytosine, which was also equal to the mC/C ratio at each reference cytosine. The function is shown as follows:

$$\mathrm{Methylation}\;\mathrm{level}=100\;*\frac{reads\;which\;covered\;methylcytosine}{effective\;sequencing\;reads}$$

Moreover, distinction between methylated and unmethylated genes is mainly whether the gene body and its 2k upstream is methylated or not. We calculated the number of reads, the number of CG sites, and the average methylation rate in each gene elements. The function of average methylation level is showed as following:

$$\mathrm{Average}\;\mathrm{methylation}\;\mathrm{level}=100\;*\frac{\sum reads\;which\;covered\;methylcytosine}{\sum effective\;sequencing\;reads}$$

Additionally, the frequencies of CHG and CHH were calculated and showed as logo plots. Methylated cytosine is in the fourth position. Furthermore, DNA methylation levels of different functional regions were performed on three different levels: chromosome, gene region and genomic feature. One cytosine was identified a effectively covered cytosine when this cytosine’s effective sequencing depth is no less than 1. Effective coverage was determined by dividing the number of effectively covered cytosine by the total cytosine in the corresponding region.

$$\mathrm{Effective}\;\mathrm{coverage}\;\mathrm{of}\;\mathrm{one}\;\mathrm{region}=100*\frac{Effectively\;covered\;cytosines}{total\;cytosines}$$

### Gene ontology enrichment analysis

Gene ontology (GO) annotations of Tibetan chicken genes were downloaded from the Ensembl genome browser (ftp://ensembl.org/pub/current/otherdata/Gene_ontology/gallus_gallus_glean_gene.go). GO comparative analyses between gene groups of interest were performed using BGI WEGO (http://wego.genomics.org.cn/cgi-bin/wego/index.pl).

### KEGG pathway analysis

Different genes usually interact with each other to exercise their biological functions. Kyoto Encyclopedia of Genes and Genomes (KEGG) is a public database useful for linking genes to their biological functions. Super geometry analyses were conducted to find the KEGG pathways enriched in genes that were differentially methylated in comparison to the whole genome. The KEGG calculation formula is the same as that in GO function analyses, where “N” represents the number of genes with pathway annotation and “n” is the number of differentially expressed genes corresponding to N; M represents number of all genes with a particular pathway annotation and “m” represents numbers of differentially expressed genes with a particular pathway annotation. Pathway mapped with q values ≤ 0.05 are defined as the pathways with significant enrichment. These pathways can then be studied to determine their biochemical role.

## Results

### Global mapping of DNA methylation

In the present study, blood samples from three Tibetan chickens were used to generate three libraries for genome-wide methylation sequencing. All libraries showed nearly complete bisulfite conversion at 99.7%. In total, 41.3 Gb of raw data were obtained from the three blood samples. After data filtering, 151,345,614, 165,745,108 and 141,554,972 clean reads were generated for the three libraries. Of the total reads, 75.6% were mapped to the reference genome, with 28 times the whole-genome average depth of coverage, which could reveal quantity of clean data (Tables 1 and 2).

**Table 1:** Statistics of data generation.

Sample	Library	Insert Size (bp)	Conversion rate (%)	Read length (bp)	Clean Reads	Clean data (Gbp)
T1	ZANxgdHACDEAAPEMI-1	325	99.7	90	151,345,614	13.6
T2	ZANxgdHACDEBAPEMI-3	360	99.7	90	165,745,108	14.9
T3	ZANxgdHACDECAPEMI-7	340	99.7	90	141,554,972	12.7

**Table 2:** Read alignment.

Sample	Raw reads (M)	Raw data (Gb)	Mapped reads (M)	Average map rate (%)	Whole genome average coverage depth (X)
T1	151	13.6	114	75.5	9
T2	166	14.9	121	72.9	10
T3	141	12.7	112	79.4	9

Cytosine patterns have 3 major types, including CG, CHG, and CHH, where “H” represents non-G bases, according to the sequence context. Therefore, we analyzed the relationships between effective sequencing depth and genome coverage for different cytosine patterns (S1 and S2 Figs). S1 Fig reveals that there is a negative correlation between the effective sequencing depth and the percentage of cytosines in the genome. S2 Fig shows that the distribution of genome coverage varies with sequencing depth according to the Poisson distribution and that the depth of the distribution`s apex is close to the genome’s average sequencing depth.

In addition, we performed effective coverage analysis based on three different levels, including the entire chromosome, the gene region, and genomic features. The effective coverage of all cytosines in each chromosome ranged from 82.77% to 97.86%, except for 24.96% in chr17. CpG effective coverage for each chromosome ranged from 86.74% to 97.5%, except for 23.58% in chr17 (S1 Table). Moreover, coverage of all cytosine in the coding regions (CDS) and intron regions were 95.94% and 93.66%, respectively, and CG coverage in CDS and intron regions were 96.04% and 93.45%, respectively (S2 Table).

### DNA methylation patterns

**Fig 1:**
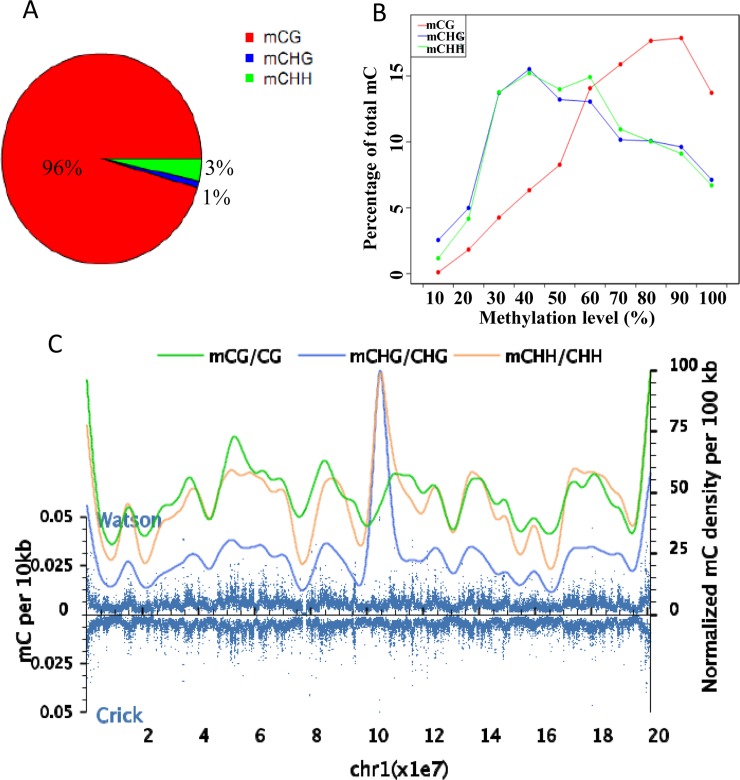
Global DNA methylation profile of the Tibetan chicken. (A) proportion of each mC type in whole genome, and the sum of proportion of three mC types is equal 100% (the symbol “%” was abridged) (B) Distribution of methylation levels for CG, CHG, and CHH. The y-axis indicates the fraction of all mCs while the x-axis represents methylation level of methyl cytosine. (C) Methyl cytosine density distribution throughout chromosome 1. Blue dots indicate methyl cytosine density in 10-kb windows throughout the chromosome. Smooth lines represent the mC density.

In Tibetan chickens, the methylation levels of all genomic C sites were more than 3.9%. Patterns of cytosine methylation in Tibetan chickens were found to have three major types, including mCG, mCHG, and mCHH, according to the sequence context. We discovered genome-wide levels of 51.22% CG, 0.4% CHG, and 0.45% CHH methylation in the Tibetan chickens (Table 3). In the whole genome, the CG methylation occupied over 96% of cytosine methylation, which is normally the primary cytosine methylation pattern. However, the rate of mCHH was only 3% and the rate of mCHG was 1% (Fig 1A).

**Table 3:** Average methylation levels for C, CG, CHG, and CHH.

Pattern	C	CG	CHG	CHH
Methylation level (%)	3.92	51.22	0.4	0.45

The methylation status of CG, CHG, and CHH differ between species and even vary within a single organism under different conditions such as time, space, and physiology. Fig 1B showed that the percentage of methyl cytosine varies with the methylation level. In the Tibetan chicken blood, more than 75% of the mCG sites were 60 to 100% methylated (Fig 1B). In addition, chromosome 1 was used to highlight the methyl cytosine density distribution in a chromosome. The methyl cytosine density showed large variation throughout chromosome 1 and this was similar to that seen in other chromosomes (Fig 1C).

### Proximal sequence features analysis

**Fig 2:**
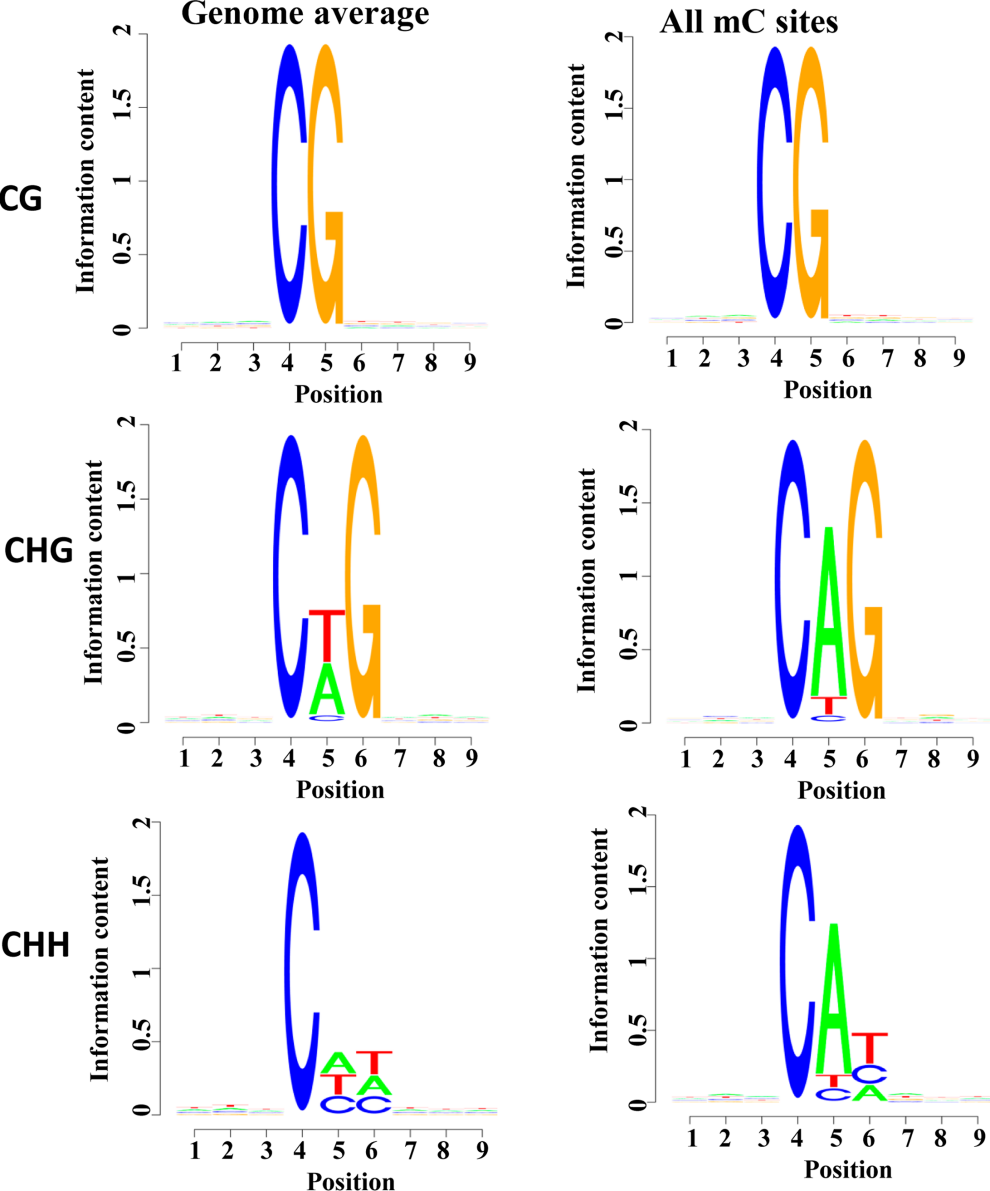
CG and non-CG proximal sequence features. Logo plots of the sequences proximal to sites of CG, CHG, and CHH DNA methylation in each sequence context.

To identify whether certain local sequences were markedly enriched as in the DNA methylome of Arabidopsis, we analyzed the sequence adjacent to sites of CG and non-CG for methylation. The methylation ratios of all potential 9-mer sequences were calculated and the methylated cytosine was located at the fourth position in these sequences. As shown in Fig 2, hardly any sequence preference was found in the CG-flanking regions of the whole genome or in the mCG-flanking regions. Moreover, the highest frequency base that was located next to the CHG cytosine in genome was A, followed by T and C. However, the base following the mCHG methyl cytosine was preferentially a T, followed by A and C. In the CHH context, the fifth position from the cytosine was preferentially a C, and the sixth position was a T, similar to the mCHH (Fig 2).

### DNA methylation levels in different functional regions

**Fig 3:**
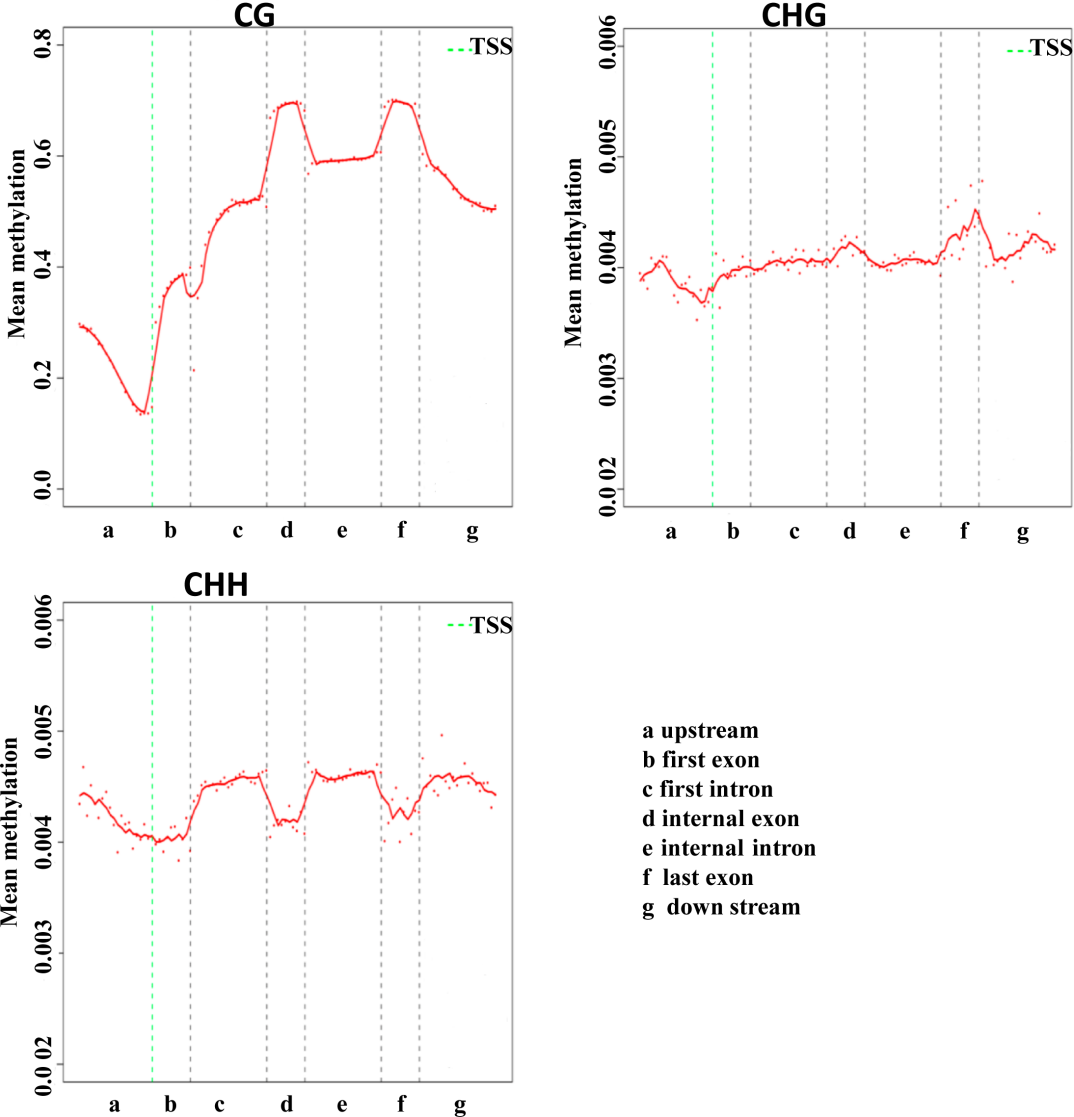
Average methylation level in different gene regions. Each dot denotes the mean methylation level per bin, and the respective lines denote the 5-bin moving average.

Different genomic features are associated with distinct regulatory functions. To study the DNA methylation profile in different genomic features, a heat map was used to present the distribution of methylation levels in the CDS, downstream region, throughout the genome, introns, and upstream regions (Fig 3). The comparative analysis of mean DNA methylation levels revealed that different genome regions showed distinguishing DNA methylation levels. Additionally, we analyzed DNA methylation patterns across the transcriptional units at the whole genome level. In Tibetan chickens, most of the promoter regions have an association with hypomethylated CpG islands. These showed a lower CG methylation level than the gene body or regions downstream of genes. Moreover, methylation of CG declined sharply before the transcription start site (TSS) and increased markedly towards the gene body regions and plateaued until the 3’ end of the gene body. There were two obvious CG methylation peaks in the internal exons and the last exon (Fig 3). The methylation of CHG had the same varying tendency as seen with the methylation of CG, but was characterized by mitigatory changes compared to the rapid changes in CG methylation. Furthermore, the methylation peaks of both CG and CHG were presented in the internal exons and last exons, where the methylation of CHH is lower.

### DNA methylation levels of promoter and gene body

**Fig 4:**
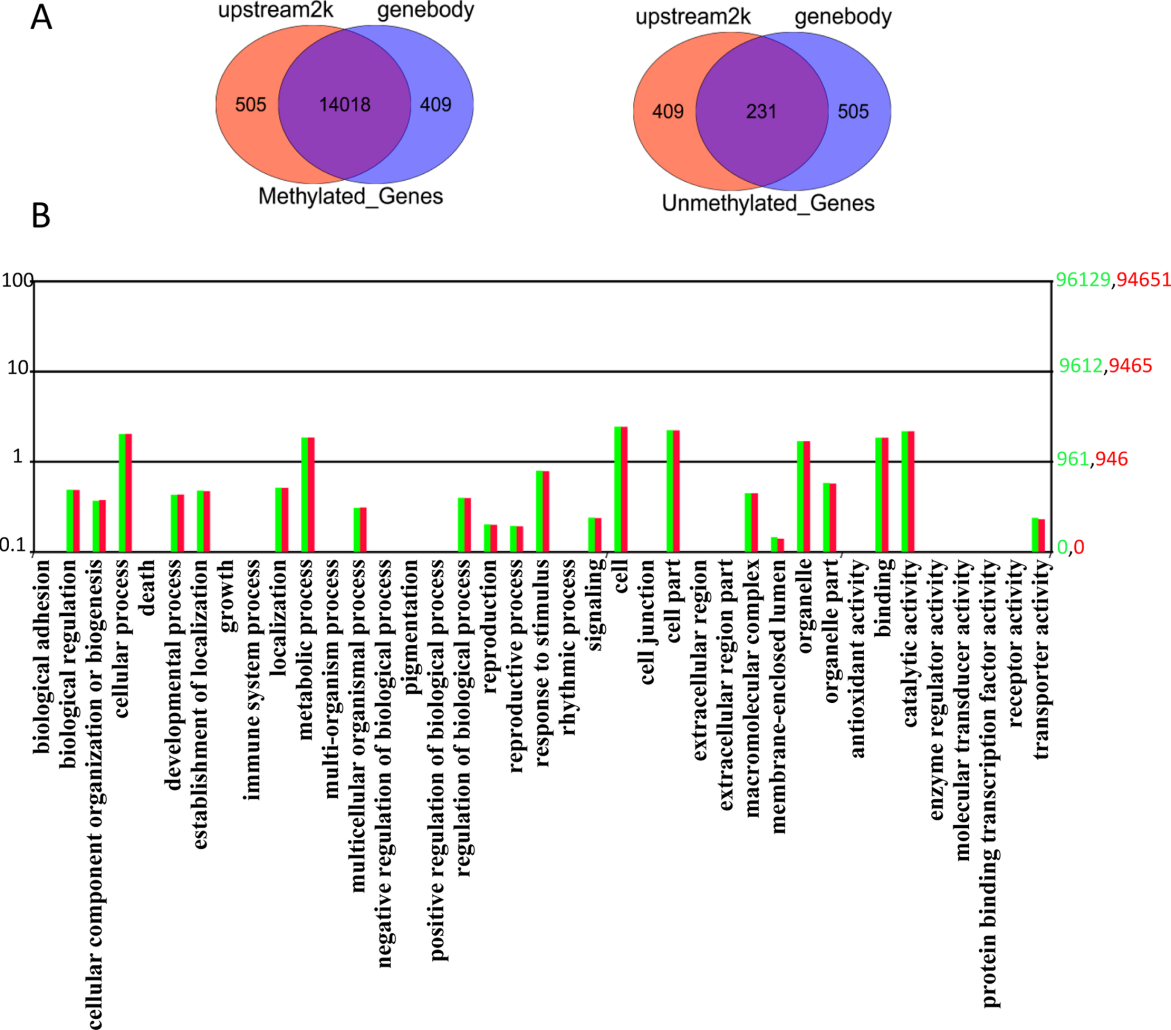
Distinguishing between methylated and unmethylated genes. (A) Number of methylated genes and unmethylated genes. (B) GO enrichment analysis for upstream methylated genes and gene body methylated genes. The x-axis indicates GO items, the left y-axis indicates the proportion of genes involved, and the right y-axis indicates the exact number of genes.

**Fig 5:**
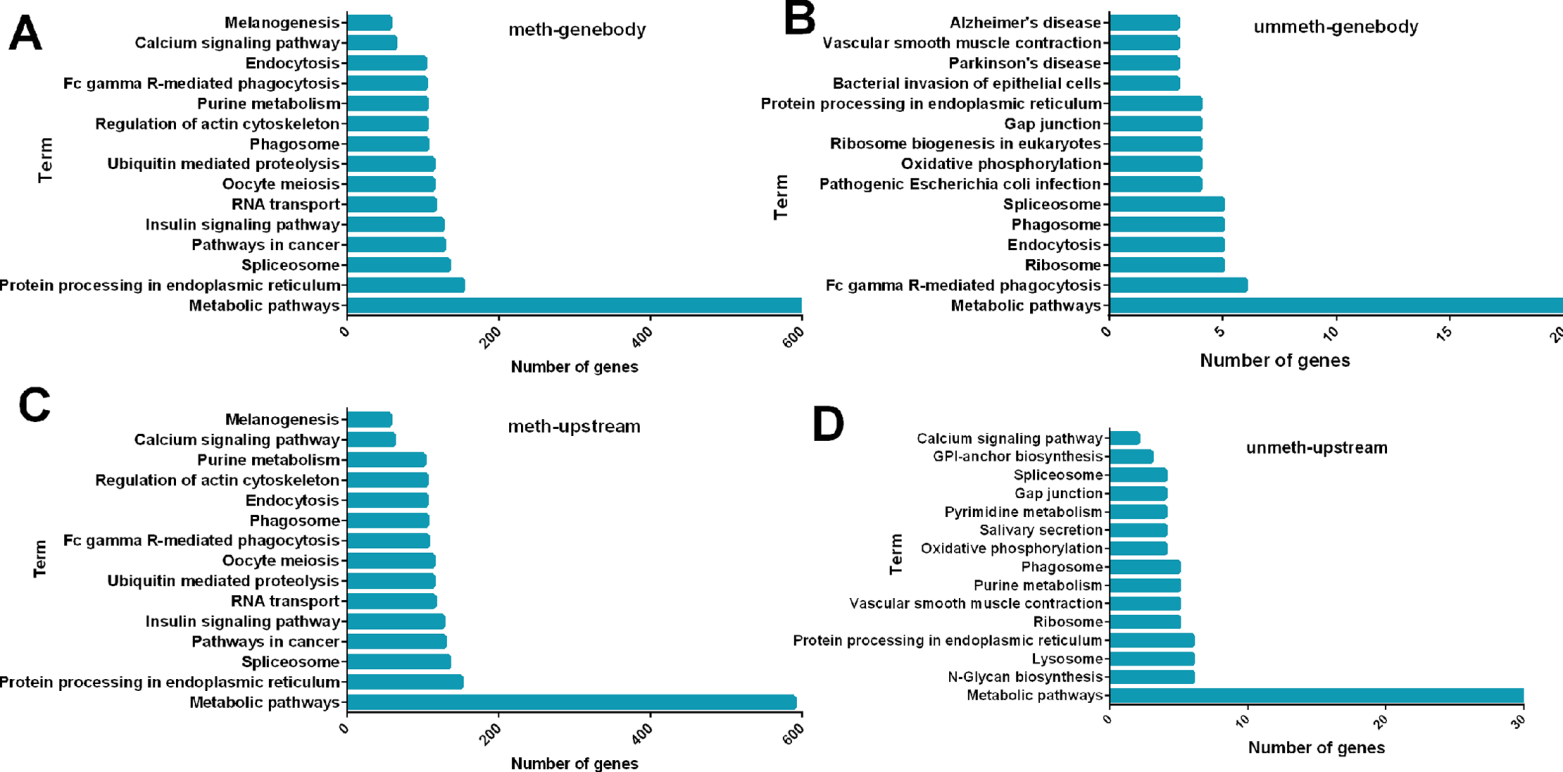
Pathway assignment based on KEGG. (A) Pathway analysis of gene body methylated genes. (B) Pathway analysis of gene body unmethylated genes. (C) Pathway analysis of upstream methylated genes. (D) Pathway analysis of upstream unmethylated genes.

Methylation of the promoter suppresses gene expression, but the functional role of gene body DNA methylation in highly expressed genes has yet to be clarified. To better characterize the methylation of the promoter and gene body, a comprehensive analysis of methylated genes and unmethylated genes in the gene body and 2k bases upstream was performed. In total, 14,018 genes were methylated in both promoter and gene body regions, while 505 and 409 genes were exclusively methylated in the promoter and gene body, respectively. There were 231 genes unmethylated in both regions (Fig 4A). GO analysis of methylated and unmethylated genes revealed the top-ranked enriched GO terms were related to cellular processes, metabolic process, and responses to stimuli in the biological process (BP) category. The cellular component (CC) category was mainly comprised of genes involved in cell, cell part, and organelle. Within the molecular function (MF) category, binding, catalytic activity, and transporter activity were highly represented (Fig 4B and S3 Fig). In addition, KEGG analysis showed that gene body methylation genes were clustered in the metabolic pathways, protein processing pathways in the endoplasmic reticulum, and calcium signaling pathways, while the gene body unmethylated genes were clustered in metabolic pathways, Fc gamma R-mediated phagocytosis, and endocytosis. Moreover, promoter methylation occurred in genes that were mostly involved in ubiquitin-mediated proteolysis, oocyte meiosis, and the formation of melanomas, while unmethylated promoters were for genes that were mostly involved in N-glycan biosynthesis, glycosylphosphatidylinositol (GPI)-anchor biosynthesis, and fat digestion and absorption (Fig 5).

### DNA methylation and gene expression levels

**Fig 6:**
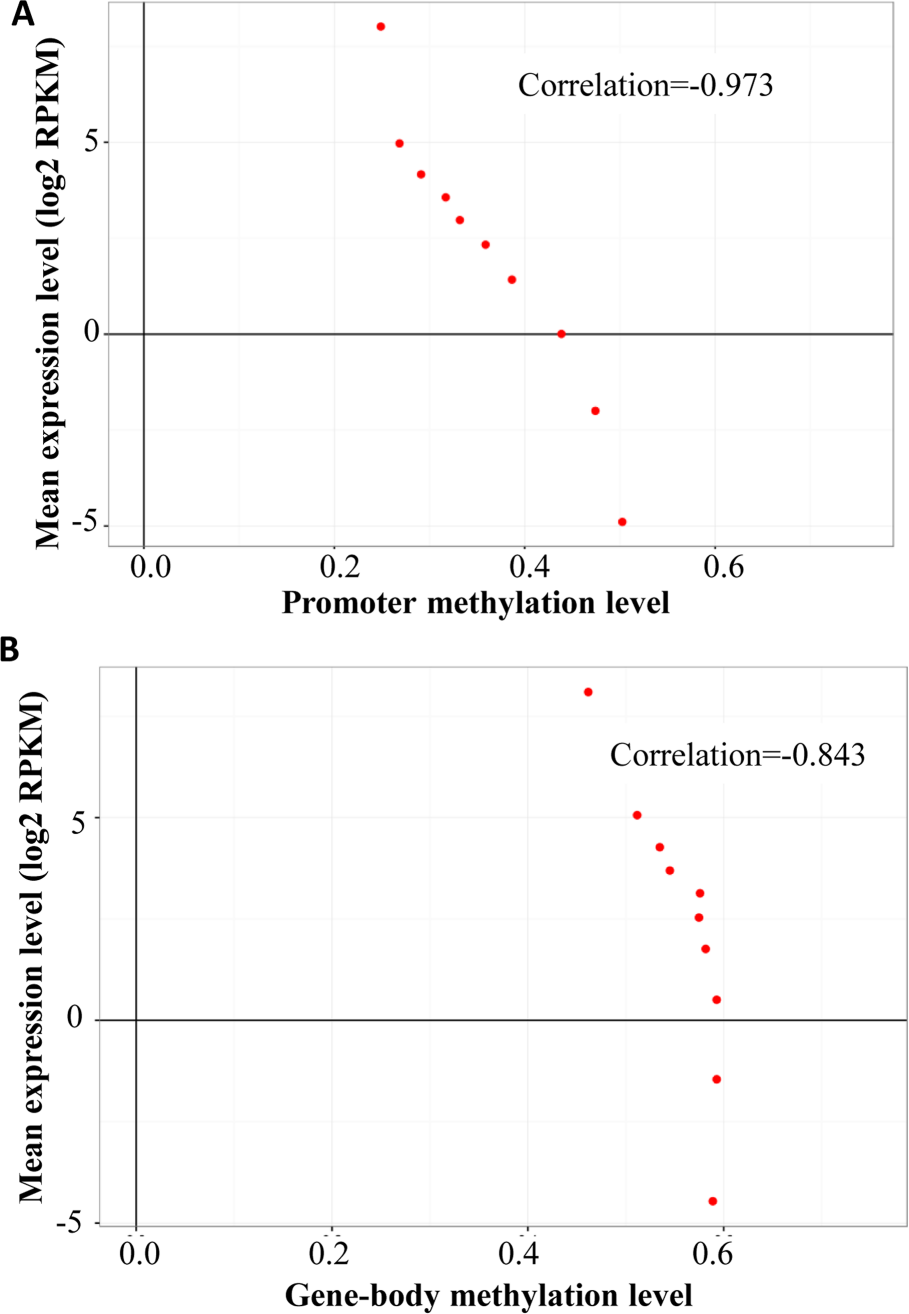
Relationship between DNA methylation and gene expression level in the Tibetan chicken. All genes were divided into ten groups according to their expression levels, from the lowest 10% and to the highest 10%. Each point represents the mean expression level and the relative methylation level. (A) Relationship between promoter DNA methylation and gene expression level. (B) Relationship between gene body DNA methylation and gene expression level.

DNA methylation of the promoter generally suppresses gene transcription via induction of a compact chromatin structure. We obtained the gene expression profiles of Tibetan chickens from the GEO database (GSE77166). Based on the expression levels, all genes were divided into ten groups, from the lowest 10% and to the highest 10%. Furthermore, the genomic regions that were 2k bases upstream of the TSS were defined as the proximal promoter and the mean methylation was used as the methylation level for each group. The correlation analysis showed that gene expression levels were negatively correlated to the mean DNA methylation levels in the promoter regions (Fig 6A; r = -0.93, p < 0.01). Unexpectedly, we found a slightly negative correlation between the gene expression levels and the gene body methylation, though the methylation showed little difference in these ten groups with respect to different expression levels (Fig 6B; r = -0.83, p < 0.01).

#### Methylation of genes involved in the adaptation to high-altitude

Using the WGBS data of lowland chicken [15], we analyzed the methylation of several candidate genes that have been identified to play key roles in the adaptation to high-altitude conditions [16]. For genes up-regulated in the highland chicken, CTGF (methylation level 0.8%), BMP4 (14.9%), ANGPTL4 (27.3%), BMP3 (0.5%), COLGALT2 (0.5%), PRKAR2B (14.1%), ETV5 (16.9%), and JSC (8.5%) were hypomethylated in the highland chicken, while their methylation levels were more than 30% in the lowland chicken. For genes with Nonsynonymous SNPs, three of them (FGFR1, JPH2 and SATB1) were hypermethylated in the highland chicken (methylation level > 70%) but were medium-methylated in the lowland chicken; one of them (SCLY) was hypomethylated in the highland chicken (methylation level 1.2%), while was medium-methylated in the lowland chicken (methylation level>30%).

## Discussion

Genomics technologies have been extensively used to investigate the adaptations of humans, animals, and plants to extreme conditions [17,18]. However, the relationships between the adaptations and the epigenetic modifications that result from extreme environmental exposures remain to be elucidated. Prior to this study, the methylation patterns of genes in Tibetan chickens was unknown. To improve our understanding of the association between epigenetic modifications and adaptations to hypoxia and high-dose ultraviolet radiation in high-altitude environments, we analyzed whole-genome and single-base resolution DNA methylomes by WGBS to provide the genome-wide DNA methylation patterns in Tibetan chicken blood and interrogated the potential role of DNA methylation in adaptation to high-altitude environments.

Genome-wide DNA methylation of lowland chickens has been investigated using MeDIP-seq [19,20], MBD-Seq [21], and Methyl-MAPS [22], which measure methylation based on immunoprecipitation and restriction enzyme digestion. Compared to WGBS, these technologies generate lower resolution and coverage and fail to obtain methylation level for CHG and CHH. For example, only 32% of CpG coverage was obtained from the study of lowland chickens using Methyl-MAPS [22]. In another lowland chicken study, the CpG coverage ranged from 83.72 to 91.57% using MethylC-seq [15]. In the current study, the effective CpG coverage of each chromosome ranged from 86.74% to 97.5%, except for 23.58% coverage of chr17.

In lowland chickens, more than 55.69% of cytosines in the CG context were methylated, which is much higher than those in the Tibetan chickens (51.22%), while the percentage of mCHG and mCHH in Tibetan chickens was higher than those in lowland chickens. In addition, 96.24%, 0.86%, and 2.89% of all methylcytosines were present in the CG, CHG, and CHH contexts of lowland chickens, respectively, while the CG methylation in Tibetan chickens occupied only 96% of cytosine methylation. Moreover, the base next to methylcytosine of mCHG in lowland chickens had a preference for A, while the preference in highland chickens was for a T. All of these indicate that chickens inhabit in different environments may have different CG methylation levels and methylation preferences, suggesting that DNA methylation may be involved in the adaptation of chickens to high-altitude environments.

In the Tibetan chicken genome, the DNA methylation levels rapidly decrease before the TSS, and after the TSS, markedly increased in the gene body region, and two obvious CG methylation peaks were present in the regions of the internal exons and the last exon. These methylation features discovered in this study consistently matched with those previously reported in bovine placentas [23]. Similar to the lowland chicken genome, the Tibetan chicken genome has two CG methylation peaks in an internal exon and last exon. However, the difference between the chicken genomes is in the region before the TSS, where there is a mitigatory methylation level in the lowland chickens[15], suggesting that Tibetan highland characteristic with long-term hypoxia and high UV radiation may cause methylation alterations in chickens.s.

The promoter plays a crucial role in the regulation of gene transcription and most of the promoter regions are usually hypomethylated [24]. Gene body DNA methylation is associated with chromatin structure and elongation efficiency, preventing spurious transcription initiation [25,26]. In the present study, we discovered that the promoter is hypomethylated, whereas the methylation level in gene body is relatively high, a finding that is similar to those previously reported in humans [27] and lowland chickens [19]. Hypermethylation of the promoters represses gene transcription [28], while a reduction of methylation at the promoters causes gene overexpression [29]. In human embryonic stem cells, Laurent et al. reported that 20% of the most highly expressed genes displayed the lowest methylation levels in promoter regions [27]. With the gene expression profiles of Tibetan chickens from the GEO database (GSE77166), we analyzed the relationship between methylation and gene expression in Tibetan chickens using the method reported in previous studies [19]. Similar to reports in humans [19,30,31] and lowland chickens [5], DNA methylation level in 2 kb upstream of genes is negatively related to the gene expression level in Tibetan chicken. This was further evidence to suggest that DNA methylation in the promoters is involved in gene silencing.

Methylation in the gene body is more prevalent than in the promoter, but its role in gene regulation remains unclear. Previous research showed that gene body methylation has an intricate correlation with expression level. Most researchers believed that methylation of the gene body is positively correlated with gene expression [27,30,32,33], although several researchers have indicated that intragenic methylation may inhibit gene transcription [25]. However, the correlation between gene body methylation and expression levels in bovine placentas is non-monotonic and moderately expressed genes show the highest methylation in the gene body [23]. Our data demonstrated that methylation in the gene body of Tibetan chickens may decrease gene expression. However, methylation in the gene body is just one of the thousands of factors that affect gene transcription. Therefore, further studies centering on DNA methylation of certain regions are needed to clarify the complicated epigenetic mechanisms underlying high-altitude environments and the relationship with adaptations to hypoxia and high-dose ultraviolet radiation.

In summary, the present study provides the first comprehensive analysis of genome-wide DNA methylation patterns in the blood of highland chickens. Our results can be used to support future studies on the epigenetic regulation in response to high-altitudes.

## Supporting information

S1 Fig Cumulative distribution of the effective sequencing depth for cytosine. The horizontal axis represents the effective sequencing depth for C, while the vertical axis represents the percentage of each kind of C at a certain sequencing depth.
(TIF)

S2 Fig Genome coverage varies with read depth. The major distribution of genome coverage varies with read depth, as shown. The horizontal axis represents sequencing depth, and the vertical axis represents the coverage ratio.
(TIF)

S3 Fig GO enrichment analysis for differential genes. The horizontal axis indicates GO items (biological process, cellular component and molecular function), the left vertical axis indicates the proportion of genes involved, and the right vertical axis indicates the exact number of genes.
(TIF)

S1 Table Effective coverage in different chromosomes. (DOCX)

S2 Table Effective coverage rate (%) in different gene regions. (DOCX)
