# Supplementary figures and images for: Whole genome bisulfite sequencing reveals unique adaptations to high-altitude environments in Tibetan chickens
Source: PLoS One. 2018 Mar 21;13(3):e0193597. doi: 10.1371/journal.pone.0193597 (PMC5862445; doi:10.1371/journal.pone.0193597)

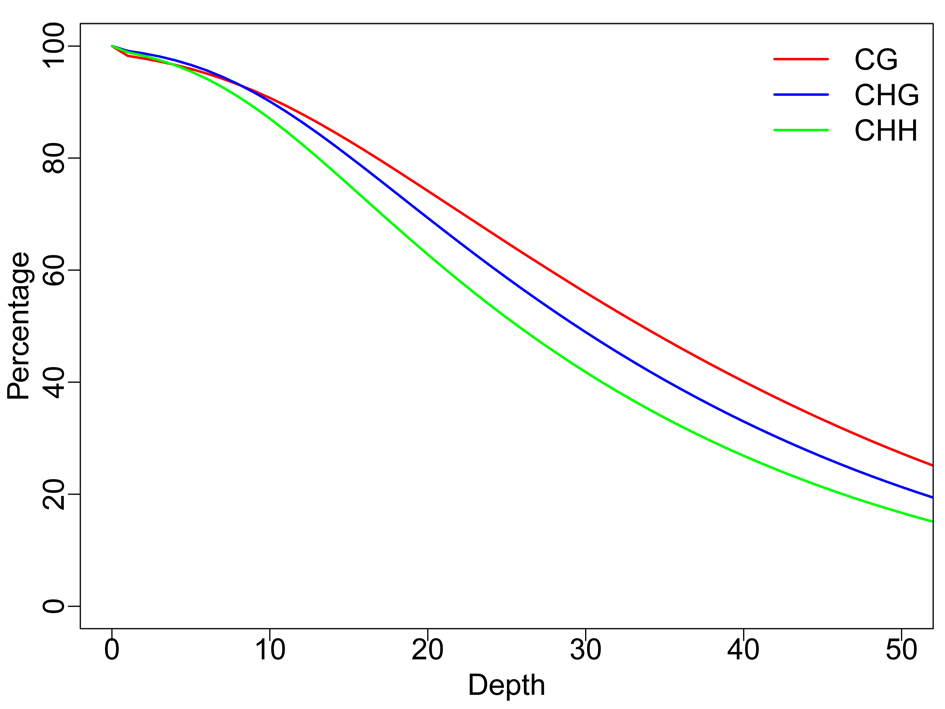

Supplement: S1 Fig — The horizontal axis represents the effective sequencing depth for C, while the vertical axis represents the percentage of each kind of C at a certain sequencing depth. (TIF) [file pone.0193597.s001.tif]

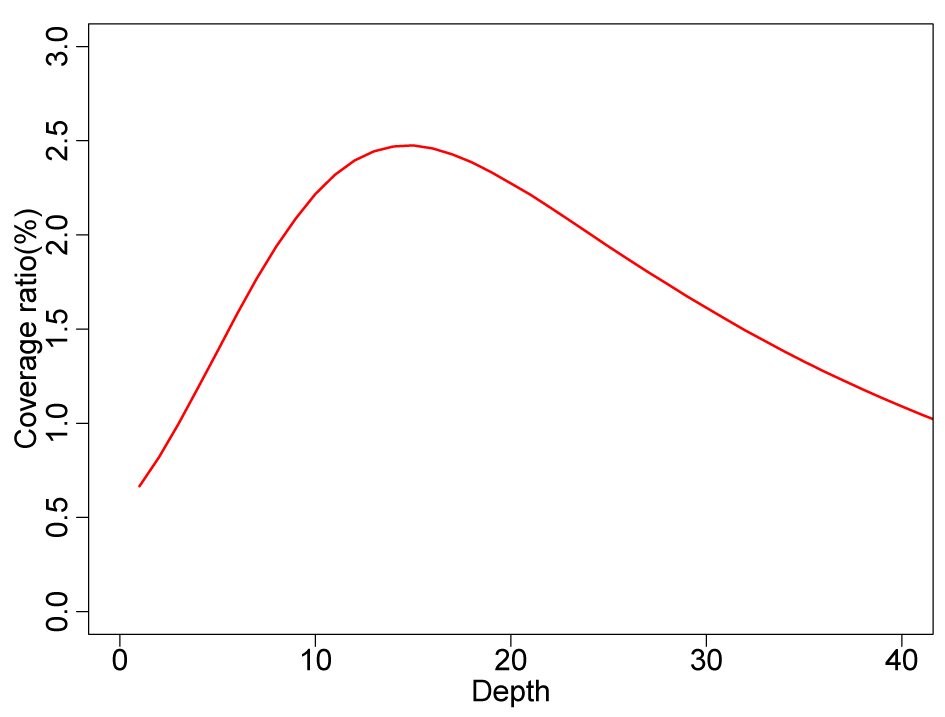

Supplement: S2 Fig — The major distribution of genome coverage varies with read depth, as shown. The horizontal axis represents sequencing depth, and the vertical axis represents the coverage ratio. (TIF) [file pone.0193597.s002.tif]

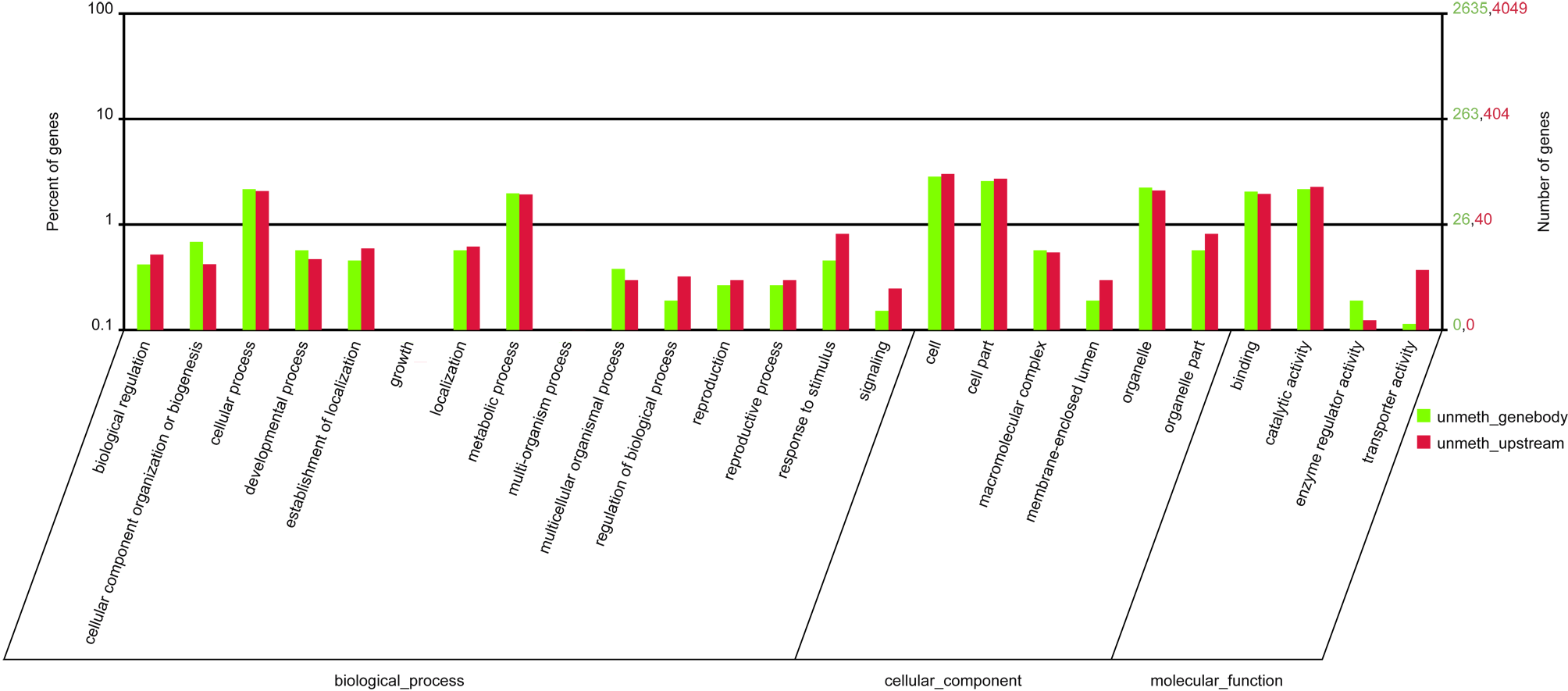

Supplement: S3 Fig — The horizontal axis indicates GO items (biological process, cellular component and molecular function), the left vertical axis indicates the proportion of genes involved, and the right vertical axis indicates the exact number of genes. (TIF) [file pone.0193597.s003.tif]
