# Supplementary material for: Whole genome bisulfite sequencing reveals unique adaptations to high-altitude environments in Tibetan chickens
Source: PLoS One. 2018 Mar 21;13(3):e0193597. doi: 10.1371/journal.pone.0193597 (PMC5862445; doi:10.1371/journal.pone.0193597)
Supplement: S1 Table — (DOCX) [file pone.0193597.s004.docx]

Table S1 Effective coverage in different chromosomes

|  | C | CG | CHG | CHH |
| --- | --- | --- | --- | --- |
| chr1 | 93.87 | 93.99 | 95.16 | 93.44 |
| chr2 | 93.56 | 93.57 | 94.83 | 93.15 |
| chr3 | 95.72 | 95.7 | 96.84 | 95.35 |
| chr4 | 94.75 | 94.99 | 95.88 | 94.36 |
| chr5 | 94.92 | 94.89 | 95.96 | 94.56 |
| chr6 | 94.66 | 94.68 | 95.69 | 94.29 |
| chr7 | 95.93 | 95.91 | 96.86 | 95.6 |
| chr8 | 96.5 | 96.4 | 97.36 | 96.21 |
| chr9 | 95.58 | 95.81 | 96.56 | 95.21 |
| chr10 | 91.9 | 92.86 | 93.11 | 91.37 |
| chr11 | 88.55 | 90.3 | 89.93 | 87.92 |
| chr12 | 96.02 | 95.94 | 96.9 | 95.7 |
| chr13 | 92.99 | 93.05 | 93.92 | 92.63 |
| chr14 | 93.5 | 94.07 | 94.55 | 93.03 |
| chr15 | 96.05 | 95.81 | 96.87 | 95.75 |
| chr16 | 82.77 | 86.74 | 85.64 | 81.1 |
| chr17 | 24.96 | 23.58 | 25.76 | 24.8 |
| chr18 | 97.47 | 97.06 | 98.34 | 97.18 |
| chr19 | 97.86 | 97.5 | 98.63 | 97.59 |
| chr20 | 97.07 | 96.76 | 97.92 | 96.77 |
| chr21 | 94.16 | 94.87 | 95.41 | 93.59 |
| chr22 | 96.14 | 95.84 | 97.07 | 95.82 |
| chr23 | 97.07 | 96.57 | 98.06 | 96.73 |
| chr24 | 95.88 | 95.72 | 96.89 | 95.49 |
| chr25 | 92.63 | 93.11 | 94.44 | 91.83 |
| chr26 | 97.48 | 97.32 | 98.44 | 97.09 |
| chr27 | 91.65 | 91.23 | 93.23 | 91.06 |
| chr28 | 96.68 | 96.28 | 97.9 | 96.23 |
| chrE22C19W28_E50C23 | 89.76 | 91.49 | 91.67 | 88.82 |
| chrE64 | 93.92 | 96.81 | 97.84 | 92.27 |
| chrMT | 82.86 | 90.75 | 90.86 | 80.34 |
| chrW | 92.54 | 93.57 | 94.39 | 91.89 |
| chrZ | 91.09 | 91.08 | 93.56 | 90.27 |
