# Supplementary material for: Whole genome bisulfite sequencing reveals unique adaptations to high-altitude environments in Tibetan chickens
Source: PLoS One. 2018 Mar 21;13(3):e0193597. doi: 10.1371/journal.pone.0193597 (PMC5862445; doi:10.1371/journal.pone.0193597)
Supplement: S2 Table — (DOCX) [file pone.0193597.s005.docx]

Table S2 Effective coverage rate(%) in different gene regions

|  | C | CG | CHG | CHH |
| --- | --- | --- | --- | --- |
| CDS | 95.94 | 96.05 | 96.31 | 95.77 |
| intron | 93.66 | 93.45 | 94.68 | 93.32 |
| mRNA | 93.79 | 93.82 | 94.77 | 93.44 |
